# Multiple Roles of Grit in the Relationship Between Interpersonal Stress and Psychological Security of College Freshmen

**DOI:** 10.3389/fpsyg.2022.824214

**Published:** 2022-03-03

**Authors:** Qingsong Yang, Mengxi Shi, Dandan Tang, Hai Zhu, Ke Xiong

**Affiliations:** ^1^School of Teacher Education, Zunyi Normal College, Zunyi, China; ^2^Institute of Advanced Studies in Humanities and Social Sciences, Beijing Normal University at Zhuhai, Zhuhai, China

**Keywords:** grit, interpersonal stress, psychological security, multiple roles, college freshmen

## Abstract

Grit, as an important positive psychological quality, has rarely been studied for its role involved in the mechanism between stress and psychological security. This article explores the moderating and mediating role of grit in the relationship between interpersonal stress and psychological security of freshmen through two studies. In study 1, freshmen from several Chinese universities (*N* = 1,224) were recruited to complete a battery of questionnaire, including assessments about interpersonal stress, grit, and psychological security. The moderating effect analysis showed that grit moderated the relationship between interpersonal stress and psychological security. Specifically, grit buffered the negative effects of interpersonal stress on freshmen’s psychological security, but this effect was obvious only when the level of interpersonal stress was relatively low, and decreased when the level of interpersonal stress was high. In study 2, college freshmen from another university apart from above ones (*N* = 604) were recruited, and we verified the results of study 1 and further explored the mediating role of grit in the relationship between interpersonal stress and security. The moderating effect analysis of study 2 also verified that of study 1. The mediating effect analysis showed that interpersonal stress not only negatively predicted psychological security, but also affected psychological security through the mediation of grit. In general, grit played a mediating and moderating role in the relationship between interpersonal stress and psychological security. This study provides first-hand evidence to explain the multiple roles of grit in the relationship between interpersonal stress and psychological security.

## Introduction

With the rise of positive psychology research, grit has received a great deal of attention from psychologists as an important theme in positive psychology and has emerged as a significant predictor of individual psychology and behavior, such as academic achievement (Duckworth et al., 2007), wellbeing (Datu et al., 2018), and job performance (Musso et al., 2019). Furthermore, some researchers have analyzed the protective effect of grit in the face of risk factors on mental health (Hobfoll, 1989; Kaniuka et al., 2020; Li and Zhu, 2020). However, previous studies have failed to further elucidate the mechanism of grit’s protective effect on mental health. There are two different views on the role of positive psychology quality in stress and mental health, including the stress-buffering hypothesis and the stress-vulnerability hypothesis (Li et al., 2012). So far, little is known the mechanisms of grit is stress-buffering or stress vulnerability in risk factors and mental health.

Regarding the role of grit in mental health, previous studies focused on the protective effect of grit (Hobfoll, 1989; Kaniuka et al., 2020; Li and Zhu, 2020), which revealed the moderating effect of grit on the relationship between risk factors and mental health. Some research found that positive psychological quality also played a mediating role in the relationship between risk factors and mental health (Cheung et al., 2021). In addition to the moderating effect, it is possible that grit may also mediate association between risk factors and mental health, that is, grit may play multiple roles in this association at the same time. Therefore, the present study seeks to explore the mechanisms underlying this association between risk factors and mental health using moderating variables and mediating variables, which may help develop methods to mitigate the negative effect of risk factors on mental health.

### The Impact of Interpersonal Stress on Psychological Security

Every year, a large number of high school students enter universities and become freshmen. This transformation of identity is a pressure and challenge for them (Praharso et al., 2017), as they will face various adaptation problems, such as new interpersonal relationships and new learning styles (Arnett, 2000; Bruffaerts et al., 2018). In this condition, if some people cannot successfully deal with these pressures, which leads to various mental health problems, they have no other choices but to drop out of school (Haktanir et al., 2018). Interpersonal stress is one of the most prominent stressors for freshmen’s mental health (Li J. et al., 2021). Interpersonal stress refers to the fact that individuals exhibit specific behavioral patterns when interacting with others, resulting in repeated difficulty in interacting with others (Hayden et al., 2019). Interpersonal stress can lead to nervous and painful emotions (Locke, 2005), as well as Internet addictions (Simcharoen et al., 2018). For college freshmen, they have to end or change the previous interpersonal relationships after entering college due to distance and rebuild new interpersonal relationships (Paul and Brier, 2001). During this period, college freshmen begin to define themselves in terms of social relations and become more concerned about their social value and acceptance by others (Tian et al., 2019). Therefore, during this period, college freshmen will pay a lot of attentions to their new classmates and friends, and invest plenty of time and energy in it (Arnett, 2000). However, due to the lack of interpersonal skills for some freshmen, they are prone to encountering interpersonal problems (Segrin and Flora, 2000; Kirkpatrick and Zang, 2011). Therefore, this study regards interpersonal stress as a prominent stressor affecting freshmen’s mental health. Although relevant studies have analyzed the impact of interpersonal stress (stressors) on college students’ mental health, the study of its mechanism is still insufficient.

Presently, viewed from the national level and social level, psychological security which is an internal need for stability has been attached more importance. *Psychological security means that a person can meet the basic needs of self-protection and feel that he is psychologically sheltered* (*supported, seen*; Zotova, 2011). It is mainly manifested as a sense of certainty and control over the people or things around the environment (Cong and An, 2004). Maslow (1943) also proposed in the hierarchy of needs theory that the sense of security is one of the basic needs of human beings. This need is not only physical, but also psychological. In fact, many human behaviors are conducted to maintain psychological security (Hart, 2014). Therefore, psychological security is of great significance to human beings. Some researchers believe that psychological security is an important factor in characterizing mental health (Iliceto et al., 2020). Some researchers even regard psychological security and mental health as synonyms (Geng et al., 2021). There are two main sources of psychological security, one is the cognition of whether the environment is safe and the other is the judgment of whether one is capable of coping with changes (Aranzamendez et al., 2014). When college freshmen shift from a familiar environment to a new environment, it forces them to face the pressure of readjusting to the new environment and rebuilding interpersonal relationship, and they are vulnerable to psychological security problems (Hiester et al., 2009; Li X. et al., 2021). Therefore, this study uses psychological security as an indicator of freshmen’s mental health. According to the theory of emotional security, an individual’s interpersonal troubles will have an impact on his psychological security (Cummings and Miller-Graff, 2015). A good interpersonal relationship is an important way to obtain a sense of security (Bostrom et al., 2013). Existing empirical studies have also shown that classmate relationship and teacher-student relationship in high school are significant predictors of psychological security of students in Grade 10 (Huang et al., 2020). Therefore, the study tried to hypothesize the following:

*Hypothesis 1*: Interpersonal stress will significantly predict psychological security.

### The Moderating Effect of Grit

Previous studies have found that interpersonal stress of college students is a risk factor affecting their mental health (Wang and An, 2014; Ye et al., 2021); however, not all people who encounter interpersonal risk factors present with mental health and behavioral problems (Forrest-Bank, S et al., 2014). The key reason lies in the protective effect of positive psychological qualities (such as grit and psychological capitals; Hobfoll, 1989; Kaniuka et al., 2020). With the rise of positive psychology orientation in the field of mental health research, the positive effects of grit on individual psychology and behavior have attracted the attention of many researchers. Grit, as a positive psychological quality, is often described as a personality characteristic for coping with difficulties and stress (Stoffel and Cain, 2018). Grit is the persistence of interest in the pursuit of goals and the perseverance to overcome difficulties and pressures (Duckworth et al., 2007; Duckworth, 2016), which mainly includes perseverance (reflecting the degree of a person’s unremitting pursuit of goals and positive altitude in facing difficulties) and passion (reflecting the degree of a person’s devotion or concentration to a certain thing or activity; Duckworth et al., 2007). Based on China’s national conditions, Lu et al. (2012) further expanded the connotation of grit on the basis of perseverance and passion, adding control (the ability to control one’s own emotions and surrounding events), and challenge (whether an individual can take change as a challenge and move forward bravely) as its factors (Lu et al., 2012). This proposal has been promoted in China, and its reliability and validity have been well verified (Yu et al., 2014; Zhang and Liang, 2016).

In recent years, researchers have paid more attentions to the protective effect of grit on mental health. According to the theoretical model of the relationship between grit and health (Maddi and Khoshaba, 1994), the protective effect of grit on mental health is mainly through moderating effect on stress, so as to maintain and improve mental health level. Some empirical studies have also verified the protective effect of grit in the face of stress on mental health. For example, Marie et al. (2019) found that grit protected college students from suicidal thoughts by weakening the negative effects of post-traumatic stress disorder (Marie et al., 2019). Li J. et al. (2021) found that grit buffered the negative effects of peer bullying and school disengagement on problematic Internet game use in adolescents (Li and Zhu, 2020). Kleiman et al. (2013) found that grit protected people from suicidal intention by increasing their pursuit of the meaning of life (Kleiman et al., 2013). Based on previous studies, this study posits that grit could buffer the impact of interpersonal stress on freshmen’s psychological security, that is, grit plays a moderating role in interpersonal stress and psychological security.

Although the protective effect of positive psychology (resilience, self-resilience, and emotional regulation) on mental health has been recognized by many researchers (Hobfoll, 1989; Vanderbilt-Adriance and Shaw, 2008; Sleijpen et al., 2017; Weissman et al., 2021), there are two different views on the mechanism of positive psychology between stress and mental health (Li et al., 2012). One is the stress-buffering hypothesis. According to this model, positive psychology can buffer the adverse effects of stress on mental health, and this buffering effect is more obvious when stress is high (Johnson et al., 2011). The theoretical model is supported by relevant empirical studies. For example, Li X. et al. (2021) found that grit buffered the negative effects of peer bullying and school disengagement on problematic Internet game use in adolescents, and the result was more obvious when peer bullying and school disengagement were serious (Li and Zhu, 2020). The other is the stress-vulnerability hypothesis, which holds that positive psychology has limited effects on mental health by buffering stress. Comparatively speaking, the buffering effect of positive psychology is greater when stress stays in low level (Hankin et al., 2004; Ingram and Luxton, 2005). This view is also supported by empirical studies. For example, previous study found that the protective effect of mental resilience on adolescents’ Internet addiction decreased with the increase of autocratic parenting style (Liu and Li, 2017). Although a series of studies were conducted on the protective effect of grit, the mechanism of this protective effect is still unclear. This study further analyzes whether the protective effect of grit is a hypothesis of stress-buffering or stress vulnerability. *It is known that, for Chinese people, interpersonal relationships are regarded as extremely important social resources and have important effects on individual psychology and behavior* (Datu, 2017). *Usually, when individuals encounter severe interpersonal stress, they will greatly lose energy and attention* (Herman, 1992), *resulting in a lack of self-confidence in the individual’s control of the surrounding environment. Therefore, the study tried to hypothesize the following:*

*Hypothesis 2*: Grit will act as a moderator between interpersonal stress and psychological security. Specially, when the level of interpersonal stress is low, grit can better protect the psychological security of college freshmen; when the level of interpersonal stress is high, the protecting effect will decline.

### The Mediating Effect of Grit

Grit not only may play a moderating role in the relationship between interpersonal stress and sense of security, but also may play a mediating role. According to the relationship model between psychological diathesis and mental health (Wang and Zhang, 2012), psychological diathesis is an endogenous factor determining the level of individual mental health, and external pathogenic risk factors will play a role through internal psychological quality. The internal psychological diathesis can not only moderate the influence of external risk factors on individual mental health, but also exert direct or mediating effect on individual mental health. Different from other personality theories, positive psychology believes that grit is not a stable personality trait, but a state trait that is influenced by a variety of factors and conducive to the healthy development of individuals (Duckworth and Yeager, 2015). According to trauma theory, it is difficult for people to focus their energy and attention on practical behaviors when facing interpersonal problems (Herman, 1992). Some researchers believe that grit act as more “state-like” than “trait-like,” which is easily changed by other factors (Jiang et al., 2019). Relevant empirical studies have also found that adverse childhood experiences would have a negative impact on the grit of college students, and different types of adverse childhood experiences would have different impacts on grit (Cheung et al., 2021). Students’ anxiety about math will weaken their grit in math learning, thus impacting math performance (Yu et al., 2021). Jiang et al. (2019) found through a longitudinal study that students’ academic performance would affect their grit level in the later period (Jiang et al., 2019). Based on previous studies, this study infers that interpersonal stress, a severe stressor for college freshmen to adapt to a new environment, negatively predicts their grit level.

In addition, as a positive personality trait, grit is also closely associated with mental health. Many relevant studies have been carried out in the field of empirical research. For example, the higher the level of grit was, the lower was the level of post-traumatic stress disorder in medical care providers (Musso et al., 2019) and the higher was the wellbeing in adults (Datu et al., 2018; Li et al., 2018), and the lower was the depression and anxiety of college students (Musumari et al., 2018). *Therefore, the study tried to hypothesize the following:*

*Hypothesis 3*: Interpersonal stress will significantly predict grit.

*Hypothesis 4*: Grit will act as a mediator between interpersonal stress and psychological security.

### Present Study

*This is helpful to better understand the role of grit in the relationship between interpersonal stress and psychological security, and to provide some ideas for future psychological security interventions. Therefore, this research sought to examine the multiple roles of grit in the relationship between interpersonal stress and psychological security from moderating variables and mediating variables, guided by the theoretical model of the relationship between psychological quality and mental health*. Study 1 explored the moderating effect of grit on the relationship between interpersonal stress and psychological security. On the basis of verifying the results of study 1, study 2 further explored the mediating role of grit in the relationship between interpersonal stress and psychological security.

## Study 1

The main purpose of study 1 was to explore whether grit plays a moderating role in the relationship between interpersonal stress and psychological security, and to further examine whether the nature of this moderating role is stress-buffering or stress vulnerability.

### Materials and Methods

#### Participants and Procedure

A total of 1,317 freshmen were recruited from colleges and universities in City Zunyi, China. After eliminating 93 incomplete questionnaires, 1,224 valid questionnaires were preserved, with an effective rate of 92.9%. The mean age of the subjects was 19.91 years old (*SD* = 0.87 years). Of these, 55.3% were girls. The entire set of questionnaires composed of the instructions and questionnaires was distributed in the form of a paper questionnaire manual and tested by participants anonymously in the classroom and collected on the spot. This study was approved by the Review Committee of Zunyi Normal College, and participants of the study voluntarily participated in this questionnaire survey.

### Measures

#### Grit

Grit was assessed using Hardiness Scale (HS) which was developed by Lu et al. (2012). There were 27 items in total, including four subscales. They are about perseverance, control, commitment, and challenge. Sample item used to measure persistence goes as “If I have a definite goal, I will not give up even when I encounter obstacles.” Sample item used to test control factors goes as “I like to try new and exciting things.” Sample item to test engagement factor goes as “I get involved in even simple tasks.” Sample item to test the challenge factor goes as “When I encounter difficulties, I always try to find a solution.” The scale is scored in four levels, with four options from 1 to 4 being “completely inconsistent,” “somewhat consistent,” “consistent,” and “completely consistent.” The higher the score is, the stronger is the personality grit. The reliability and validity of this questionnaire have been fully verified (Yu et al., 2014). In this study, reliability coefficients (Cronbach’ α) of the internal consistency of subscales involving perseverance, control, commitment and challenge, and total scale were 0.76, 0.83, 0.81, 0.79, and 0.94, respectively. In this study, the total score of the questionnaire is used to describe grit, mainly because the total score of the questionnaire is considered to be a comprehensive evaluation of grit (Duckworth and Gross, 2014; Li et al., 2018).

#### Interpersonal Stress

Interpersonal stress was assessed using the Interpersonal Relation Synthetic Diagnose Test (Zheng, 1999), which has 28 items in total, including four factors: conversation, making friends, manner of dealing with people and events, and heterosexual communication. “Yes” counts for 1 point and “no” counts for 0 point. The higher scores indicate severe interpersonal stress. In this study, reliability coefficients (Cronbach’ α) of the internal consistency of subscales involving conversation, making friends, manner of dealing with people and events, and heterosexual communication and total scale were 0.73, 0.78, 0.65, 0.71, and 0.90, respectively. For a brief description of interpersonal stress, the subsequent data are presented with a total score for interpersonal stress.

#### Psychological Security

Psychological security was assessed using Psychological Safety Scale (Cong and An, 2004). There are 16 items in total, including two main factors of interpersonal security and certainty in control. The scale was scored at five levels, with five options from 1 to 5 being “very consistent,” “basically consistent,” “neutral or uncertain,” “basically inconsistent,” and “very inconsistent.” The higher the total score is, the stronger is the person’s psychological security. In this study, the reliability coefficients (Cronbach’ α) of internal consistency of subscale involving interpersonal security and certainty in control and total scale were 0.88, 0.88, and 0.93, respectively. In order to briefly describe psychological security, the subsequent data are expressed as the total score of psychological security.

#### Covariates

*As socioeconomic characteristics, such as age and gender, have a significant impact on the total grit scores* (*See* Kannangara et al., 2018)*, we treated the socioeconomic characteristics of the subjects as covariates, in order to get the true relationship between grit, interpersonal stress, and psychological security*, such as age and gender (1 = male; 2 = female), annual family income (1 = less than 10,000 yuan, 2 = 10,000–30,000 yuan, 3 = 30,000–60,000 yuan, 4 = 60,000–100,000 yuan, 5 = more than 100,000 yuan), only child (1 = only child, 2 = non-only child), and household registration (1 = rural, 2 = city).

#### Data Analysis

SPSS 26.0 was used for data analysis, and all missing values were removed from the data. Harman’s single-factor test was used to check the degree of deviation of the common methods in this study. Unrotated exploratory factor analysis of all variables showed that there were 13 factors with characteristic roots greater than 1, and the variance explained by the first factor was 20.95%, which did not exceed the critical standard of 40%. Therefore, it can be assumed that there was were no serious common method biases in the variables involved in Study 1. Descriptive analysis and correlation analysis were adopted to test the mean and standard deviation of each variable and their correlation coefficients. Then, hierarchical regression analysis was used to examine the moderating effect of grit on the relationship between interpersonal stress and psychological safety, and the significance of moderating effects was further conducted by a simple slope test. In addition, all these variables, such as age, gender, family income, birth, and only child of college freshmen, were treated as covariates.

### Results and Discussion

Means, standard deviations, and intercorrelations among all variables are presented in Table 1. As expected, interpersonal stress was significantly negatively correlated with psychological security (*r* = −0.62, *p* < 0.001). Grit significantly negatively correlated with interpersonal stress (*r* = −0.21, *p* < 0.001) and positively correlated with psychological security (*r* = 0.36, *p* < 0.001).

**Table 1:** The mean (*M*), standard deviation (*SD*), and correlations of main variables in Study 1.

S. No.		*M*	*SD*	1	2	3
1.	Interpersonal stress	8.32	6.19	–		
2.	Grit	66.03	12.43	−0.21^***^	–	
3.	Psychological security	52.05	12.33	−0.62^***^	0.36^***^	–

*N* = 1224,

*** *p* < 0.001.

Hierarchical regression analysis was used to examine the moderating effect of grit on the relationship between interpersonal stress and psychological security, and the results showed that interpersonal stress significantly negatively predicted psychological security (*β* = −0.56, *p* < 0.001; Hypothesis 1), while grit significantly positively predicted psychological security (*β* = 0.24, *p* < 0.001). The interaction effect of interpersonal stress and grit significantly predicted psychological security (*β* = −0.07, *p* < 0.01), suggesting that grit had a significant moderating effect, as shown in Table 2.

**Table 2:** Analysis of the moderating effect of grit on interpersonal stress and psychological security in Study 1.

Variables	*F*	*В*	*SE*	*β*	*t*
Step 1	3.63^**^				
Age		0.49	0.41	0.03	1.18
Sex		−2.19	0.73	−0.09	−2.99^**^
Annual family income		0.59	0.31	0.06	1.89
Only child		−1.54	1.27	−0.04	−1.22
Household registration		0.62	1.26	0.02	0.49
Step 2	134.34^***^				
Interpersonal stress		−1.12	0.04	−0.56	−25.36^***^
Grit		0.24	0.02	0.24	10.86^***^
Step 3	119.71^***^				
Interpersonal stress × grit		−0.01	0.01	0.07	−3.20^**^

** *p* < 0.01,

*** *p* < 0.001.

**Figure 1:**
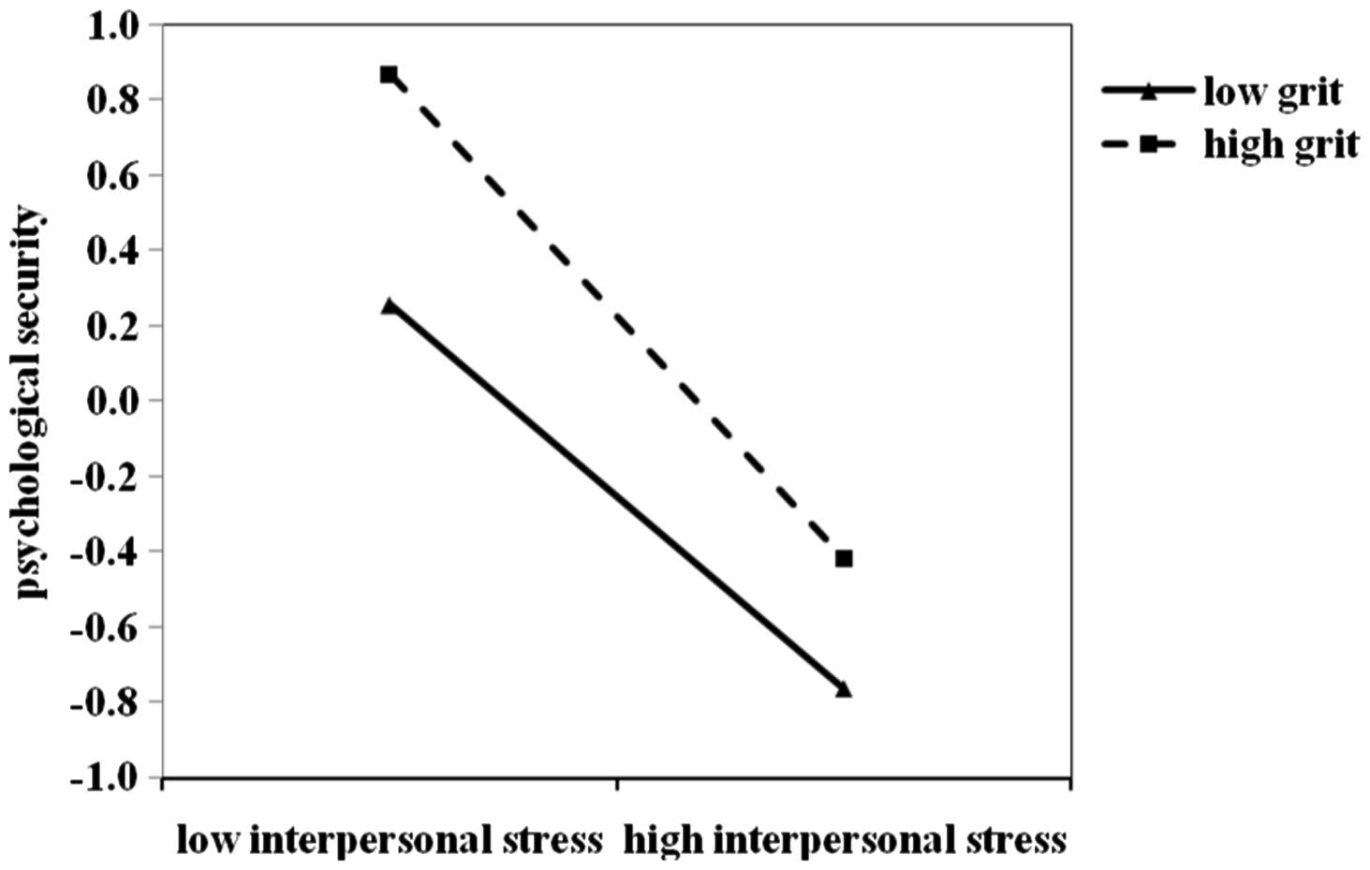
The moderating role of grit on the relationship between Interpersonal stress and Psychological security in Study 1. All the study variables were standardized. High/low level of Interpersonal stress = M ± 1SD. High/low level of grit = M ± 1SD.

In order to better present the essence of the moderating effect of grit on the relationship between interpersonal stress and psychological security, high score group and low score group were divided based on the mean value of grit plus or minus one standard deviation, and a simple effect analysis chart was drawn, as shown in Figure 1. Simple slope test found that when the grit level was low (*M*-1*SD*), psychological security showed a declining trend with interpersonal stress score increases (simple slope = −0.51, *t* = −18.36, *p* < 0.01); with the increase of interpersonal stress score, the decreasing trend of psychological security was more obvious when grit level was high (simple slope = −0.64, *t* = −19.42, *p* < 0.01). In other words, with the increase of interpersonal stress, the protective effect of grit on psychological security decreased rapidly. Therefore, the moderating effect of grit on the relationship between interpersonal stress and psychological security could be explained by the stress-vulnerability model (Hypothesis 2).

## Study 2

First, study 2 used another set of data to re-examine the stability of study 1’s results, namely, to examine the moderating effect of grit on the relationship between interpersonal stress and psychological security. Secondly, the mediating effect of grit was examined on interpersonal stress and psychological security.

### Materials and Methods

#### Participants and Procedure

A total of 647 freshmen were recruited from another university in city Guiyang, Province Guizhou, China. After 43 incomplete questionnaires were removed, 604 valid questionnaires remained, with an effective rate of 93.3%. The mean age of these was 19.93 years old (*SD* = 0.83 years), 54.3% were girls. Its procedure was the same as that of study 1.

#### Measures

Questionnaires covering grit, interpersonal stress, and psychological security were the same as these used in study 1, and intrinsic reliability was recalculated using the data collected from Study 2. The reliability coefficients (Cronbach’ α) of the internal consistency of subscales involving perseverance, control, commitment and challenge, and total scale were 0.76, 0.85, 0.81, 0.80, and 0.95, respectively. The reliability coefficients (Cronbach’ α) of internal consistency of the internal consistency of subscales involving conversation, making friends, manner of dealing with people and events, and heterosexual communication and total scale were 0.74, 0.78, 0.69, 0.73, and 0.91, respectively. The reliability coefficients (Cronbach’ α) of internal consistency of subscale involving interpersonal security and certainty in control and total scale were 0.89, 0.88, and 0.94, respectively.

#### Data Analysis

Except for the mediating effect analysis method, the data analysis tools and procedures used in study 2 were the same as those used in Study 1. The results of Harman’s single-factor test showed that there were 15 factors with characteristic roots greater than 1, and the variance explained by the first factor was 21.94%, which did not exceed the critical standard of 40%. Therefore, it could be assumed that there were no serious common method biases in the variables involved in Study 2. In study 2, the PROCESS Macro developed by Hayes (2018) was used to analyze the mediating effect, with interpersonal stress as the independent variable, grit as the mediating variable, and psychological security as the dependent variable. Bias-corrected bootstrapped confidence intervals were used to test the significance of the mediating effect of grit (subsample *N* = 5,000), and the regression coefficients were standardized. At the same time, the age, gender, family income, family birth, and only child of freshmen were treated as covariates.

### Results and Discussion

Means, standard deviations, and intercorrelations among all variables were presented in Table 3. As expected, interpersonal stress was negatively correlated with psychological security (*r* = −0.60, *p* < 0.001; Hypothesis 2). Grit negatively correlated with interpersonal stress (*r* = −0.22, *p* < 0.001) and positively correlated with psychological security (*r* = 0.39, *p* < 0.001).

**Table 3:** The mean (*M*), standard deviation (*SD*), and correlation coefficients of the main variables in Study 2.

S. No.		*M*	*SD*	1	2	3
1.	Interpersonal stress	8.09	6.25	–		
2.	Grit	66.39	12.94	−0.22^***^	–	
3.	Psychological security	52.51	12.55	−0.60^***^	0.39^***^	–

*N* = 604,

*** *p* < 0.001.

**Figure 2:**
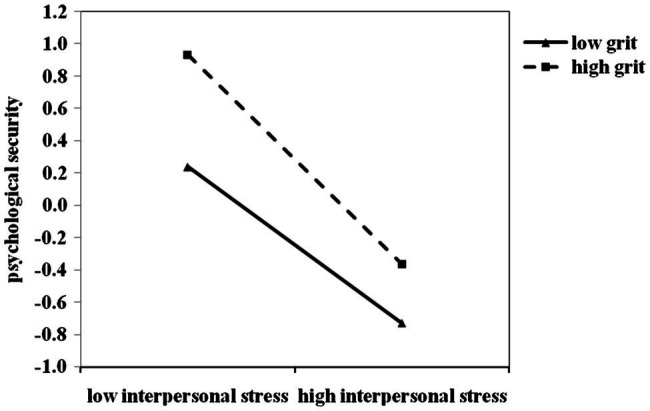
The moderating role of grit on the relationship between Interpersonal stress and Psychological security in Study 2. All the study variables were standardized. High/low level of Interpersonal stress = M ± 1SD. High/low level of grit = M ± 1SD.

Furthermore, we repeated the steps in study 1 to examine the moderating effect of grit on the relationship between interpersonal stress and psychological security. The results showed that interpersonal stress negatively predicted psychological security (*β* = −0.53, *p* < 0.001), while grit positively predicted psychological security (*β* = 0.26, *p* < 0.001). The interaction effect of interpersonal stress and grit significantly predicted psychological security (*β* = −0.09, *p* < 0.01), suggesting that grit had a significant moderating effect, as shown in Table 4. In order to better present the essence of the moderating effect of grit on the relationship between interpersonal stress and psychological security, the method of study 1 was repeated and a simple effect analysis chart was drawn, as shown in Figure 2. Simple slope analysis showed that when the grit level was low (*M*-1*SD*), the psychological security followed a decreasing trend with the increase of interpersonal stress score (simple slope = −0.48, *t* = −12.33, *p* < 0.01). With the increase of interpersonal stress score, the decrease of psychological security was more obvious when grit level was high (simple slope = −0.64, *t* = −12.86, *p* < 0.01). In other words, with the increase of interpersonal stress, the protective effect of grit on psychological security decreased rapidly (Hypothesis 2). Therefore, Study 2 again verified that the moderating effect of grit on the relationship between interpersonal stress and psychological security which could be explained by the stress-vulnerability model.

**Table 4:** Analysis of the moderating effect of grit on interpersonal stress and psychological security in Study 2.

Variables	*F*	*В*	*SE*	*β*	*t*
Step 1	2.85^**^				
Age		1.30	0.63	0.09	2.08^*^
Sex		−2.52	1.06	−0.10	−2.38^*^
Annual family income		0.48	0.45	0.04	1.06
Only child		−2.10	1.81	−0.05	−1.16
Household registration		1.62	1.76	0.04	0.92
Step 2	63.39^***^				
Interpersonal stress		−1.07	0.07	−0.53	−16.61^***^
Grit		0.25	0.03	0.26	8.14^***^
Step 3	56.98^***^				
Interpersonal stress × grit		−0.01	0.01	−0.09	−2.71^**^

* *p* < 0.05,

** *p* < 0.01,

*** p < 0.001.

**Figure 3:**
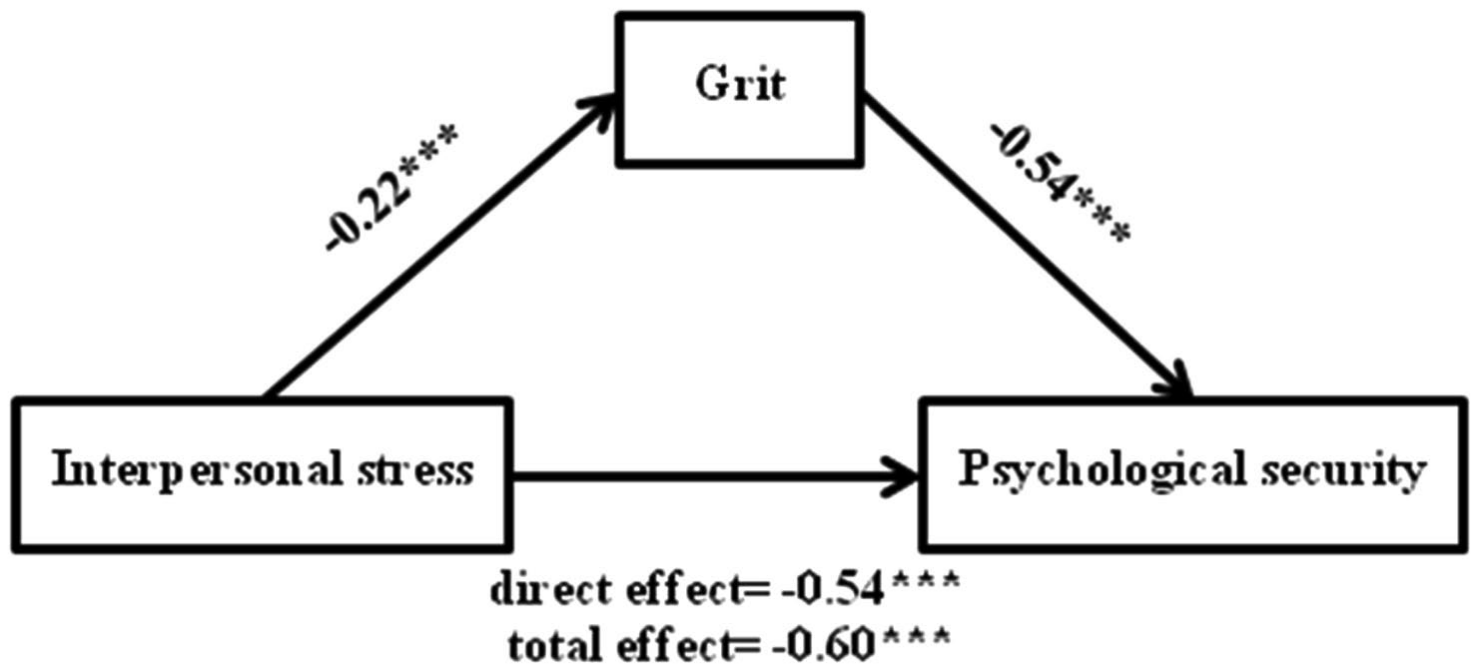
Mediation model of interpersonal stress as a predictor of psychological security mediated by grit. Standardized regression coefficients are displayed for all paths. ^***^p < 0.001.

Finally, we examined the mediating effect of grit on the relationship between interpersonal stress and psychological security. The results showed that the direct effect of interpersonal stress on psychological security was −0.54 (95% CI = [−0.60, −0.47]), and the indirect effect of grit was −0.06 (95% CI = [−0.09, −0.03]). Bootstrap 95% confidence interval for the mediating effect did not include 0, indicating that the mediating effect of grit was significant, accounting for 10% of the total effect, as shown in Figure 3. The results of this study indicated that interpersonal stress of college freshmen could directly affect the level of psychological security and also indirectly affected the level of psychological security through the mediation of grit (Hypothesis 3 and 4).

## General Discussion

Guided by the relationship model of psychological quality and mental health, this study took college freshmen as participants and explored the effecting mechanism of grit on the relationship between interpersonal stress and psychological security. All hypotheses were supported, and both study 1 and 2 found that grit could buffer the effect of interpersonal stress of college students on the level of psychological security, but this buffering only evidently be showed in the lower level of interpersonal stress, and this buffering declined rapidly at higher levels of interpersonal stress. In addition, this study also found that interpersonal stress not only directly affected the level of psychological security, but also had an impact on the level of psychological security through the mediation of grit.

### The Moderating Effect of Grit

This study found that grit played a moderating role in the relationship between interpersonal stress and psychological security, which supported the theoretical view of the relationship between grit and health (Kobasa and Puccetti, 1983). The theory described grit as a protective factor when people deal with stressful events, that is, grit could buffer the adverse effects of stress on mental health. At the same time, this result is consistent with the relevant empirical results. Marie et al. (2019) found that grit protected individuals who suffered traumatic experiences from suicidal idea. We can understand the protective role of grit against stress in this way: gritty individuals will actively seek change in the face of stress and difficulties (Duckworth et al., 2009) and mobilize their own positive emotions and resources to cope with stress, so as to alleviate the adverse effects of stress on themselves and avoid anxiety, depression, and other adverse emotions (Musumari et al., 2018). Moreover, gritty individuals are more likely to receive social support (Wallace et al., 2001). Thus, gritty individuals may exhibit less mental health confusion and higher levels of psychological security. It is worth noting that an important finding of this study is that grit had a significant buffering effect on stress only when the level of stress was low, so the protective effect of grit on psychological security is suitable to be explained by the stress-vulnerability model (Li et al., 2012). This phenomenon can also be mentioned in related research on individual positive resources. For example, Praharso et al. (2017) found that social support was also vulnerable to buffering the impact of life changes on college freshmen depression. The protective effect of grit on mental health was more obvious when the stress was low. When the pressure was high, the protective effect of grit would decrease significantly. This suggests that although grit has a protective effect on people’s mental health, we should not overstate the role and scope of grit. Therefore, in the future intervention of psychological security, it should not be ignored to focus on relieving the stressors themselves (such as interpersonal stress).

### The Mediating Effect of Grit

In this study, grit was also found to have a mediating role in the relationship between interpersonal stress and psychological security. In other words, interpersonal stress could directly affect the level of psychological security and also could affect psychological security through the indirect effect of grit. It could be explained in two ways. On the one hand, interpersonal stress negatively predicts grit. This is consistent with the findings of related studies that adverse childhood experiences negatively predict grit level of college students (Cheung et al., 2021). According to self-determination theory, the satisfaction of belongingness needs is the basic premise for a person’s healthy growth and development (Ryan and Deci, 2000). If an individual encounters interpersonal troubles, such as being rejected, ignored, or repelled, his sense of belonging will not be satisfied. This sense of belonging in interpersonal relationships is very important for Chinese people who are collectivist-value-oriented (Datu, 2017), which will affect the persistence of individual behaviors and make it difficult for individuals to focus their energy and attention on behaviors (Herman, 1992), thus leading to a decline in grit level. One the other hand, grit positively predicted psychological security. This is consistent with the findings of relevant studies that grit positively predicted the psychological security of the elderly (Wallace et al., 2001). It can be interpreted as follows: gritty individuals are more likely to pursue the meaning of life and goals in life (Hill et al., 2016), and they tend to believe that their abilities can be improved through their own efforts (West et al., 2016). They exhibit higher self-discipline in daily life and learning (Muenks et al., 2016) and self-improving learning behaviors (Wolters and Hussain, 2014), thus making their lives more fulfilling and secure.

Especially in Study 2, grit was found to be functional in mediating the relationship between interpersonal stress and psychological security, which provides implications for future interventions of grit or psychological security. On the one hand, grit is not fixed to some extent, but relatively variable and plastic (Wang et al., 2018; Alan et al., 2019), which could be affected by external or internal factors (Wong and Vallacher, 2018); therefore, intervention methods can be used in the future. For example, improving academic performance (Tang et al., 2019), expanding social support (Wallace et al., 2001), and fostering growth mindset (Wang et al., 2018) can improve the grit level of individuals (Alan et al., 2019). On the other hand, in addition to improving interpersonal relationships, grit can also serve as a starting point to improve the psychological security of college freshmen.

### The Multiple Roles of Grit

In this study, we found that grit plays multiple roles between interpersonal stress and psychological security, namely, a moderating role and a mediating role. This means that interpersonal stress indirectly affects psychological security through the mediation of grit, and the relationship between interpersonal stress and psychological security depends on the level of grit. In other words, grit is a bridge of interpersonal stress affecting psychological security, and will change the direction and intensity of interpersonal stress and psychological security, and is an important resource for college freshmen to maintain mental health. The results of this study support the view of the relationship model between psychological quality and mental health, that is, risk factors play a role through internal psychological quality, and the internal psychological quality can not only moderate the impact of external risk factors on individual mental health, but also influence individual mental health level directly or serves as a mediating variable (Wang and Zhang, 2012). The simultaneous moderating and mediating effects of one variable have been similarly verified in other studies (Dicke et al., 2014; Attar-Schwartz, 2015). *Based on the findings of this study, combined with the view of the relationship model between psychological quality and mental health* (Liu and Li, 2017)*, we propose an dual-effects theory about grit. According to this theoretical model, grit can be regarded as a complex binary construct that affects mental health. On the one hand, as a plastic variable, grit is affected by external risk factors, such as interpersonal stress. The interpersonal stress will inhibit the positive effects of grit to a certain extent. In this case, grit mediates the relations between external risk factors and mental health. On the other hand, as a relatively stable and important personality trait, grit can also interact with external risk factors to affect mental health. Thereby, grit may act as mediator and moderator between external risk factors and mental health at the same time, changing the direction and intensity of the risk factors’ impact on mental health. In general, the results of this study expand our understanding of the concept of grit, namely, grit is relatively stable and variable; at the same time, integrating with the previous research results, we consider that grit acts on as both mediator and moderator in mental health, and therefore, the present study helps us deeply understands the mechanism of grit between risk factors and mental health. Moreover, the results of this study can also help us to explain the development outcome of mental health* (*such as psychological security*) *and the complex process of its formation. Taken together, the present study provides important theoretical insights and practical guidance for both the prevention and intervention of mental health*.

### Limitations

This study has several limitations. First of all, this study conducted a cross-sectional study on the moderating and mediating effects of grit on the relationship between interpersonal stress and psychological security, and a cautious attitude should be taken in interpreting the results (Fiedler et al., 2011). This is because cross-sectional methods cannot well describe the causal relationship among interpersonal stress, grit, and psychological security. In addition, this study found that the protective effect of grit on psychological security was applicable to low level of interpersonal stress, which supported the hypothesis of stress vulnerability of positive psychological quality instead of the stress-buffering hypothesis. However, it remains to be explored whether the protective effect of grit decreased because the depletion of grit was caused by high level of interpersonal stress. In the future, longitudinal research can be used to explore the influence of different levels of interpersonal stress on grit, so as to verify whether high level of interpersonal stress will cause the loss of grit. Secondly, this study collected data through self-evaluation, and some of the questionnaires involved the evaluation of individuals, such as The Interpersonal Relation Synthetic Diagnose Test, which is easy to induce the social evaluation anxiety of the participants, and may lead to the concealment of true responses of the participants, thus interfering with the results of the study. Therefore, future studies can consider interviews or other evaluation methods (Hill et al., 1998). Thirdly, this study only analyzes the influence of interpersonal stress on grit and psychological security, without examining the influence of other possible important factors, such as students’ academic performance. Some studies showed that academic performance was closely related to grit and psychological problems of college freshmen (Bruffaerts et al., 2018). In addition, the data in this study were selected from objects with Chinese cultural background, and whether it can be extended to objects of other cultures remains to be further tested. For instance, Chinese society emphasizes collectivism, while the West emphasizes individualism (Wang et al., 2019). The question of whether interpersonal stress might have a more pronounced effect on grit in China other than in the West remains to be explored.

## Conclusion

This study examined the effecting mechanism of grit on the relationship between interpersonal stress and psychological security of college freshmen, and found that grit played multiple roles. First, grit moderated the relationship between interpersonal stress and psychological security. Specifically, grit could buffer the negative impact of interpersonal stress on psychological security. However, this effect was obvious only when the level of interpersonal stress was low, and decreased rapidly when the level of interpersonal stress was high. Secondly, grit played a mediating role between interpersonal stress and psychological security, that is, interpersonal stress not only directly affects psychological security, but also affects psychological security through the mediating role of grit.

## Data Availability Statement

The original contributions presented in the study are included in the article/supplementary material, further inquiries can be directed to the corresponding author.

## Ethics Statement

The studies involving human participants were reviewed and approved by the Committee of Zunyi Normal College. Participation was voluntary, based on written informed consent, and withdrawal from the studies was allowed at any given time. Written informed consent was obtained from all participants for the publication of any potentially identifiable images or data included in this article.

## Author Contributions

QY and KX conceived and designed the study. MS, DT, and HZ contributed to data collection. QY analyzed the data. QY, MS, and KX wrote the paper. All authors reviewed and approved the manuscript.

## Funding

This paper is supported by the National Social Science Fund of China (grant number BBX210284).

## Conflict of Interest

The authors declare that the research was conducted in the absence of any commercial or financial relationships that could be construed as a potential conflict of interest.

## Publisher’s Note

All claims expressed in this article are solely those of the authors and do not necessarily represent those of their affiliated organizations, or those of the publisher, the editors and the reviewers. Any product that may be evaluated in this article, or claim that may be made by its manufacturer, is not guaranteed or endorsed by the publisher.
